# Incidental Benign Metastasizing Leiomyoma in a Patient with Bone Sarcoma: A Case Report

**DOI:** 10.1155/2014/439061

**Published:** 2014-08-26

**Authors:** Zanndor Jacob del Real-Romo, Carlos Montero-Cantú, Oscar Villegas-Cabello, José Antonio Díaz-Elizondo, Danae Reyes-Salas, Rene Palomo-Hoil, Guillermo Peralta-Castillo, David Martínez-Sánchez, Eduardo Flores-Villalba

**Affiliations:** ^1^Escuela de Medicina y Ciencias de la Salud del Tecnológico de Monterrey-TEC Salud, Avenida Doctor Ignacio Morones Prieto 3000, Colonia Los Doctores, 64710 Monterrey, NL, Mexico; ^2^Hospital San José-TEC Salud, Avenida Doctor Ignacio Morones Prieto 3000, Colonia Los Doctores, 64710 Monterrey, NL, Mexico; ^3^Universidad Autónoma de Nuevo León, Avenida Francisco I. Madero y Avenida Gonzalitos S/N, Colonia Mitras Centro, 64460 Monterrey, NL, Mexico; ^4^Hospital Zambrano Hellion-TEC Salud, Batallón San Patricio 112, Colonia Real de San Agustin, 66278 San Pedro Garza García, NL, Mexico

## Abstract

*Background*. The benign metastasizing leiomyoma is an exceptionally rare entity; it presents with ectopic leiomyoma nodules with a benign pattern. Symptoms vary according to the anatomic location. The diagnosis is histopathological, usually in patients with history of hysterectomy. *Case Presentation*. A 36-year-old female with 2-month history of left knee pain was diagnosed with bone fibrosarcoma. A CT scan showed pulmonary nodules. The patient started neoadjuvant chemotherapy. Conservative surgery of pelvic limb was achieved. A new CT scan reported pulmonary nodules that remained in relation to the previous CT. A nodule resection by thoracotomy and TOB (transoperative biopsy) was performed. The final pathology report described benign proliferative lesions consistent with benign metastatic leiomyoma. *Conclusions*. Benign metastatic leiomyoma is a rare condition presenting with uterine and extrauterine nodules most commonly in the lung. The diagnosis is histopathological. The surgical procedure must be reserved for selected patients.

## 1. Background

The benign metastasizing leiomyoma is an exceptionally rare entity with few cases reported in the world literature; it is associated with ectopic (extrauterine) leiomyoma nodules with a benign pattern in the lungs [1]. Most patients are asymptomatic and the nodules are discovered incidentally. When symptoms do occur, they vary according to the anatomic location of the lesion [2]. The diagnosis is histopathological, usually in patients with history of hysterectomy. The treatment is hormonal, reserving surgical intervention for selected patients.

## 2. Case Presentation

**Figure 1:**
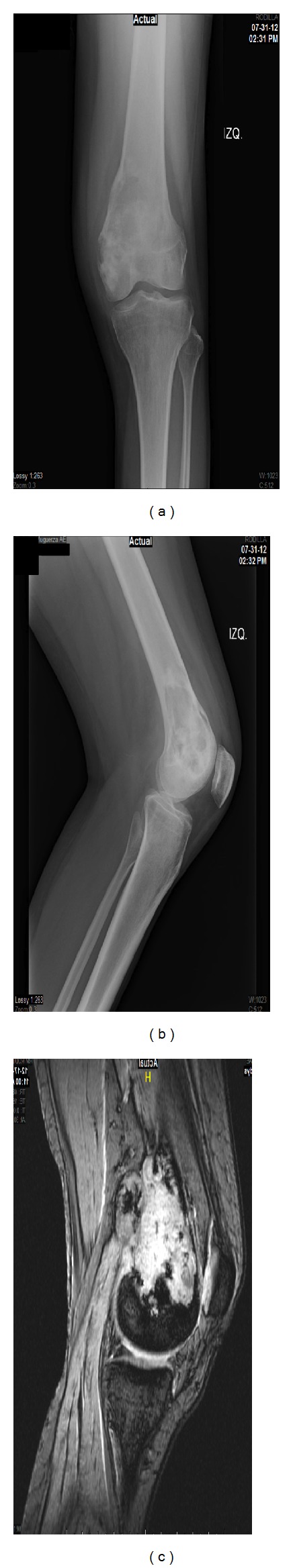
((a) and (b)) Permeative pattern in distal femur; (c) malignant looking lesion is corroborated with a MRI. MRI: magnetic resonance imaging.

**Figure 2:**
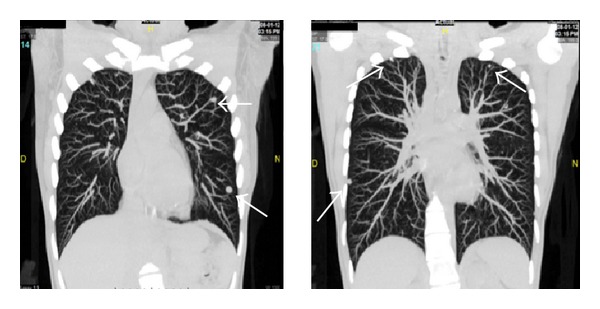
Pulmonary nodules (arrows) in both hemithoraces on a CT scan.

**Figure 3:**
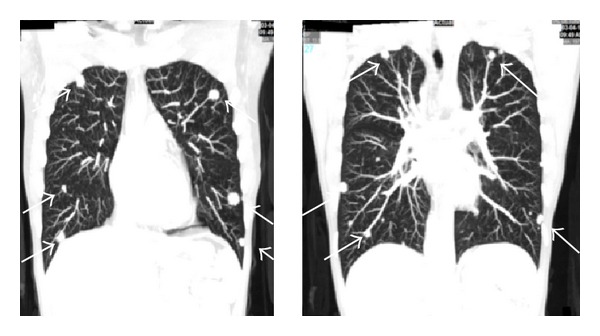
CT scan 3 months later reported multiple pulmonary nodules (arrows) that remain in number and dimension in relation to the previous CT.

**Figure 4:**
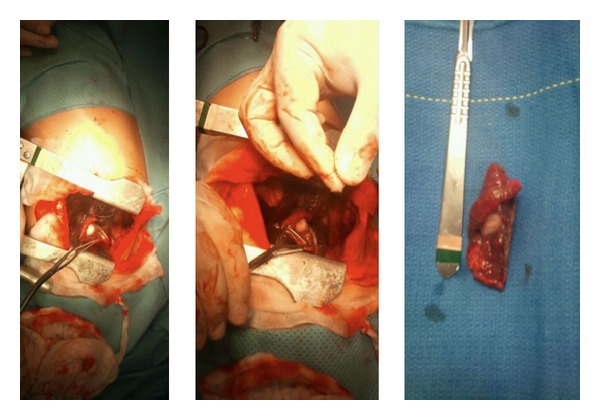
Nonanatomic pulmonary resection of superior and inferior left lobe was performed by posterolateral thoracotomy.

**Figure 5:**
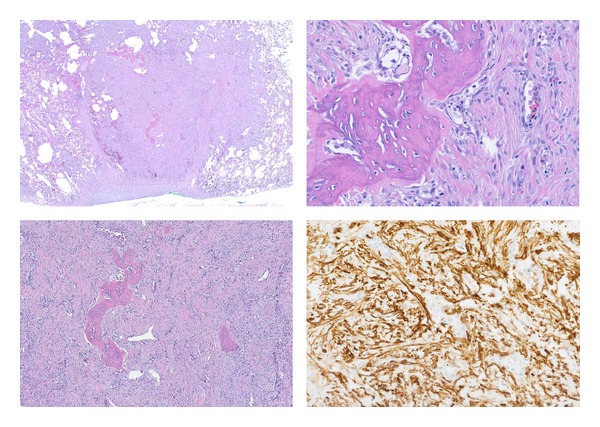
Fibromuscular tissue growth with nonneoplastic ossification; immunohistochemistry nodules composed of cells, vimentin, AML, calponin, ALK1, a240, D, and Ki67 20%. AML: actina de músculo liso (smooth muscle actin).

**Figure 6:**
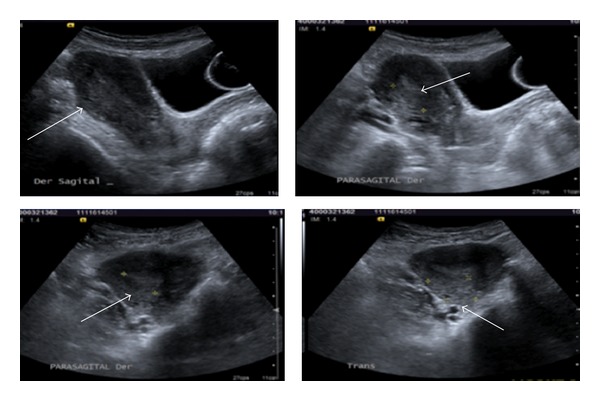
Pelvic US shows uterine myoma in posterior wall of 30 × 25 × 20 mm. Two myomas in the interior wall.

A 36-year-old multiparous Mexican woman, married, living in Monterrey, NL, with past medical history of two plastic surgeries, cesarean, and an abortion. She presents with 2-month history of left knee pain, progressive in intensity from a mild discomfort to a severe disabling pain. It increased with walking. The physical examination revealed increased volume of the knee, tenderness, and edema, the X-ray showed a permeative pattern in distal femur (Figures 1(a) and 1(b)) without evidence of fracture; the malignant-looking lesion was corroborated with a MRI (Figure 1(c)). The biopsy was consistent with fibrosarcoma. The evidence of pulmonary nodules (smaller than 1 cm) in CT scan suggested metastatic disease in both hemithoraces (Figure 2). The patient started 5 cycles of neoadjuvant cisplatin and doxorubicin, and a conservative surgery of the pelvic limb with resection of distal femur, proximal tibia, and femur prosthesis placement was achieved. Pathology diagnosis reported 100% remission of femur sarcoma after chemotherapy. Months later a CT scan demonstrated that pulmonary nodules remained in number and dimensions in relation to the previous CT scan (Figure 3). Through posterolateral thoracotomy a nonanatomic pulmonary segment resection of right superior and inferior lobes with 60 mm blue cartridge GIA stapler (Covidien Surgical, Norwalk, CT, USA) was performed (Figure 4); the transoperatory biopsy described a white nodular lesion of approximately 1 cm extension with benign pattern. The final pathology report described benign fibromuscular proliferative lesions, cells forming nodes, by immunohistochemistry, vimentin, AML, calponin, ALK1, d240, re, and 20% of Ki67, (Figure 5) consistent with benign metastasizing leiomyoma, which was corroborated by three uterine myomas in a pelvic US (Figure 6).

## 3. Discussion

Benign metastasizing leiomyoma is a rare condition characterized by the presence of extrauterine leiomyoma nodules with the lung being the most common site, although, it has also been reported in skin, pelvis, abdomen, muscle, omentum, inferior vena cava, right atrium, brain, and bone [2]; until 1996 there were 74 cases reported in the literature [1]; there are currently about 150 cases.

This condition presents with development of leiomyoma lung tumors, well-differentiated and histologically benign behavior due to its low mitotic rate, absence of nuclear pleomorphism, absence of local invasion, and indolent curse. Molecular studies suggest that this is a monoclonal process similar to ordinary leiomyomas [3]. There are some theories about vascular or lymphatic spread or venous dissemination during hysterectomy. Some animal studies suggest that the expansion to other tissues is by haematogenous dissemination, intraperitoneal implantation, or coelomic metaplasia [3, 4].

The disease is asymptomatic and often discovered incidentally; if symptoms are present they would be chest pain, dyspnea, and cough. Pathological and immunohistochemical studies are necessary to exclude lymphangioleiomyomatosis; benign metastasizing leiomyoma is characterized by having less, smaller, and more limited interstitial lung and visceral muscle destruction [2]. The histological analysis suggests that pulmonary nodules arise from benign uterine lesions after a hysterectomy as treatment for leiomyomas. Due to the expression of estrogen and progesterone receptors, the treatment is based on GnRH agonists, selective receptor modulators of estrogens, aromatase inhibitors, or progestins; oophorectomy can also be considered [1]. Hysterectomy is not necessary for extrauterine disease, although some masses were reported with low-grade uterine sarcoma [4, 5]. The largest series was reported by Kayser et al.; most of the patients had median survival of 43 months after lung biopsy and did not die of benign metastasizing leiomyoma [6].

## 4. Conclusions

There are variants of clinically and histologically benign uterine leiomyomas. In particular, benign metastasizing leiomyoma is a rare condition with ectopic nodules of uterine leiomyoma in the lung. The diagnosis is histopathological after an incidental finding since its presentation is asymptomatic. The gold standard treatment is based on hormonal therapy. The surgical procedure must be reserved for selected patients.

## References

[1] Jautzke G, Müller-Ruchholtz E, Thalmann U (1996). Immunohistological detection of estrogen and progesterone receptors in multiple and well differentiated leiomyomatous lung tumors in women with uterine leiomyomas (so-called benign metastasizing leiomyomas): a report on 5 cases. *Pathology Research and Practice*.

[2] Egberts J, Schafmayer C, Bauerschlag DO, Jänig U, Tepel J (2006). Benign abdominal and pulmonary metastasizing leiomyoma of the uterus. *Archives of Gynecology and Obstetrics*.

[3] Patton KT, Cheng L, Papavero V (2006). Benign metastasizing leiomyoma: clonality, telomere length and clinicopathologic analysis. *Modern Pathology*.

[4] Awonuga AO, Shavell VI, Imudia AN, Rotas M, Diamond MP, Puscheck EE (2010). Pathogenesis of benign metastasizing leiomyoma: a review. *Obstetrical and Gynecological Survey*.

[5] Yoon G, Kim T-J, Sung C-O (2011). Benign metastasizing leiomyoma with multiple lymph node metastasis: a case report. *Cancer Research and Treatment*.

[6] Kayser K, Zink S, Schneider T (2000). Benign metastasizing leiomyoma of the uterus: documentation of clinical, immunohistochemical and lectin-histochemical data of ten cases. *Virchows Archiv*.

